# The Impact of LY487379 or CDPPB on eNOS Expression in the Mouse Brain and the Effect of Joint Administration of Compounds with NO^•^ Releasers on MK-801- or Scopolamine-Driven Cognitive Dysfunction in Mice

**DOI:** 10.3390/molecules29030627

**Published:** 2024-01-29

**Authors:** Agata Płoska, Anna Siekierzycka, Paulina Cieślik, Lawrence W. Dobrucki, Leszek Kalinowski, Joanna M. Wierońska

**Affiliations:** 1 Department of Medical Laboratory Diagnostics—Fahrenheit Biobank BBMRI.pl, Medical University of Gdansk, 7 Debinki Street, 80-211 Gdansk, Poland; agata.ploska@gumed.edu.pl (A.P.); anna.siekierzycka@gumed.edu.pl (A.S.); dobrucki@illinois.edu (L.W.D.); 2 Maj Institute of Pharmacology Polish Academy of Sciences, 12 Smetna Street, 31-343 Krakow, Poland; pa.cieslik@gmail.com; 3 Department of Bioengineering, University of Illinois at Urbana-Champaign, Urbana, IL 61801, USA; 4 Beckman Institute for Advanced Science and Technology, Urbana, IL 61801, USA; 5 Department of Biomedical and Translational Sciences, Carle-Illinois College of Medicine, University of Illinois at Urbana-Champaign, Urbana, IL 61801, USA; 6 BioTechMed Center, Department of Mechanics of Materials and Structures, Gdansk University of Technology, 11/12 Narutowicza Steet, 80-223 Gdansk, Poland

**Keywords:** mGlu2, mGlu5, eNOS, DETANONOate, spermineNONOate, MK-801, scopolamine, novel object recognition

## Abstract

The role of endothelial nitric oxide synthase (eNOS) in the regulation of a variety of biological processes is well established, and its dysfunction contributes to brain pathologies, including schizophrenia or Alzheimer’s disease (AD). Positive allosteric modulators (PAMs) of metabotropic glutamate (mGlu) receptors were shown to be effective procognitive compounds, but little is known about their impact on eNOS expression and stability. Here, we investigated the influence of the acute and chronic administration of LY487379 or CDPPB (mGlu2 and mGlu5 PAMs), on eNOS expression in the mouse brain and the effect of the joint administration of the ligands with nitric oxide (NO) releasers, spermineNONOate or DETANONOate, in different combinations of doses, on MK-801- or scopolamine-induced amnesia in the novel object recognition (NOR) test. Our results indicate that both compounds provoked eNOS monomer formation, and CDPPB at a dose of 5 mg/kg exaggerated the effect of MK-801 or scopolamine. The coadministration of spermineNONOate or DETANONOate enhanced the antiamnesic effect of CDPPB or LY487379. The best activity was observed for ineffective or moderate dose combinations. The results indicate that treatment with mGluR2 and mGluR5 PAMs may be burdened with the risk of promoting eNOS uncoupling through the induction of dimer dissociation. Administration of the lowest possible doses of the compounds with NO^•^ donors, which themselves have procognitive efficacy, may be proposed for the treatment of schizophrenia or AD.

## 1. Introduction

Nitric oxide (NO) is a gaseous neurotransmitter biosynthesized endogenously through the oxidation of nitrogen during conversion of L-arginine to L-citrulline in the presence of cofactors, such as NADPH and tetrahydrobiopterin (BH_4_) [1]. Under normal conditions, the reaction is mediated by two NO synthases, endothelial and neuronal (eNOS and nNOS). eNOS-derived NO exerts anti-inflammatory and proangiogenic effects, modulates the expression and processing of amyloid precursor protein (APP) in cerebrovascular endothelium and neuronal tissues [2], and thus regulates many aspects of brain homeostasis, such as blood–brain barrier (BBB) permeability, protein folding and vasodilation [3]. nNOS-derived NO is crucial in the formation of the glutamate–NO–cGMP axis and is essential in the regulation of long-term potentiation (LTP), a process critical in learning and memory [4]. Both NO synthases exert their physiological roles as dimers [5].

Pathological conditions such as NOS uncoupling with its cofactors, depletion of L-arginine, or disruption of NOS dimers into monomers result in the formation of superoxide (O_2_^•−^) instead of NO [6,7]. O_2_^•−^ may contribute to the generation of other reactive oxygen species (ROS), but also further reacts with NO, producing peroxynitrite (ONOO^−^), a highly toxic form of reactive nitrogen species (RNS), leading to the production of other secondary components of nitroxidative stress, such as NO_2_^+^, NO_2_ and OH^•^ [8]. These processes initiate a cascade of redox reactions, deleterious neuroimmune signals and toxic neuroinflammatory responses, reduced cerebral perfusion, impaired homeostatic processes in the cerebral microenvironment, and interactions between brain innate and peripheral adaptive immunity, which contribute greatly to the cognitive and behavioral symptoms of schizophrenia [6,7,9].

Endothelial dysfunction may also favor the onset and progression of atherosclerosis, vasoconstriction and impaired cerebral blood flow regulation and may promote neurodegeneration. Chronic loss of eNOS results in increased amyloid precursor protein level, increased amyloid beta formation and microglial activation, which result in cognitive decline and cardiovascular dysfunction related to Alzheimer’s pathology [10,11,12,13,14,15].

Considering these dynamics, the impact on eNOS expression is one of the most important factors in developing new potential strategies for the treatment of AD or schizophrenia [16,17,18,19,20,21,22,23]. For years, metabotropic receptors for glutamate have been regarded as potent antipsychotic or anti-Alzheimer’s agents [16,17,18,19,20,21,22,23], and a huge attempt has been made to introduce mGlu ligands into the clinic. Despite a number of encouraging results, some obstacles still appear that prevent enthusiasm towards mGlu receptor ligands [24,25,26,27]. There is limited knowledge on the impact of the compounds on the neurovascular unit, eNOS expression and the related putative detrimental effects.

Among all subtypes of mGlu receptors, mGlu_2_ and mGlu_5_ in particular have been studied as potential targets for novel antipsychotic and, to a lesser extent, anti-Alzheimer’s drugs [28,29,30,31]. The mGlu_5_ receptor is expressed postsynaptically and is linked with guanylate cyclase, which further produces cGMP, activating an intracellular signaling cascade [31], while mGlu_2_ receptors, expressed presynaptically on nerve terminals, are negatively linked with adenyl cyclase activity, and their activation inhibits glutamate release [32,33,34]. These properties make them excellent targets to treat CNS disorders. However, their impact on eNOS expression and simultaneous action with NO donors have not been investigated.

In the present studies, the influence of positive allosteric modulators of mGlu_5_ (CDPPB) and mGlu_2_ (LY487379) receptors on eNOS expression in pharmacologically driven models of cognitive decline were examined. Similar to previous research, MK-801 was used to induce schizophrenia-related cognitive symptoms and scopolamine was used to induce Alzheimer’s-type dementia [35,36]. Subsequently, the efficacy of the simultaneous activation of mGlu receptors and NO release in novel object recognition (NOR) were examined.

## 2. Results

### 2.1. Compounds and Experimental Design

Table 1 contains all essential information about the compounds used in the present research.

In all our experiments, MK-801 was administered at a dose of 0.3 mg/kg and scopolamine at 1 mg/kg [35,36,41,42,43]. The doses of spermineNONOate and DETANONOate were established in our previous investigations [35,36]. The doses of CDPPB and LY487379 on MK-801-induced deficits were adjusted from [41,42,43]. The dose-dependent studies on the activity of CDPPB and LY487379 on scopolamine-induced deficits in NOR were performed in the present research.

Western blotting:

- Acute administration at active doses:
  − LY487379—1 mg/kg or CDPPB (5 mg/kg) with MK-801 (0.3 mg/kg);
  − LY487379—1 mg/kg or CDPPB (2 mg/kg) with scopolamine (1 mg/kg).

The frontal cortex (FC) and hippocampus from each animal were dissected 30 min after administration.

- Chronic administration for 14 days at low and top doses.
  − LY487379—0.1 or 1 mg/kg; CDPPB—0.1 and 5 mg/kg with MK-801 (0.3 mg/kg);
  − LY487379—0.1 or 1 mg/kg; CDPPB—0.5 and 2 mg/kg with scopolamine (1 mg/kg).

The FC and hippocampus from each animal were dissected 24 h after the last administration.

Novel object recognition (NOR):

- Dose-dependent studies for LY487379 and CDPPB on scopolamine-induced dysfunction. The compounds were administered at the following doses: LY487379—0.1, 0.5 and 1 mg/kg; CDPPB—0.5, 1 and 2 mg/kg.
- The activity of simultaneous administration of ineffective, moderately effective and top doses of CDPPB or LY487379 with NO^•^ releasers: slow NO releaser DETANONOate or fast releaser, spermineNONOate, on MK-801- or scopolamine-induced cognitive deficits. The scheme of administration was thought to resemble, to some extent, an isobolographic scheme of analysis. The exact doses are summarized in Table 2.

The compounds, alone or in combinations, were administered 30 min before MK-801 or scopolamine, which were administered 30 min before the T1 session (for a detailed description, please see the Section 4).

The appropriate solvents were administered instead of compounds in controls, MK-801- or scopolamine-treated animals. The solvents had no influence on the studied factors. 

### 2.2. eNOS Expression

**Figure 1:**
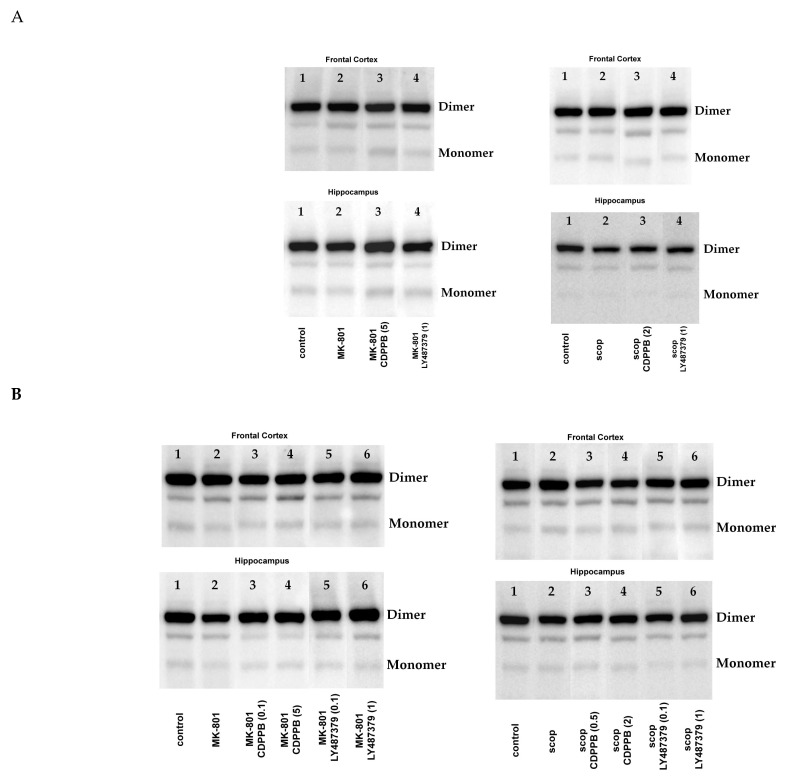
Representative blots for Western blot results. Acute administration (**A**) and chronic administration (**B**) of MK-801 or scopolamine with investigated compounds. Each line corresponds to the treatments from Figure 1, Figure 2A, Figure 3 and Figure 4B. Dimers ~250 kDa and monomer ~130 kDa.

The amount of eNOS monomer, dimer/monomer (D/M) ratio and monomer/total protein (M/T) ratio were calculated for each blot. The representative blots are presented in Figure 1.

#### 2.2.1. Acute Administration

**Figure 2:**
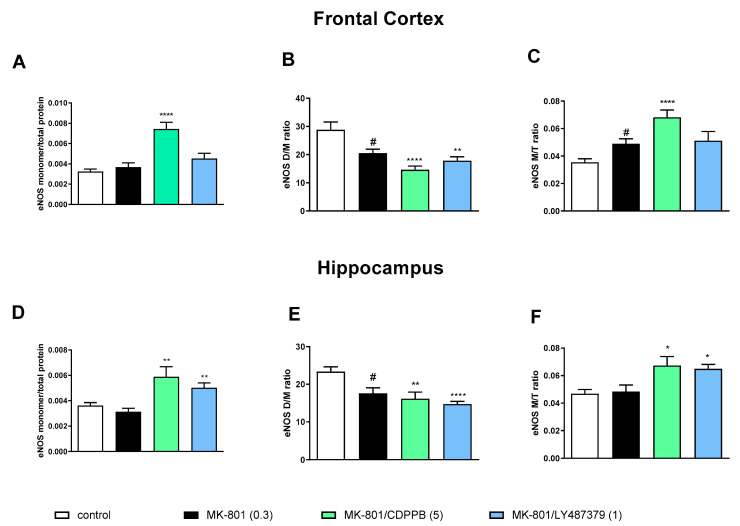
The impact of acute administration of MK-801, CDPPB and LY487379 on eNOS monomer (M) content, eNOS dimer (D)/monomer (M) protein ratio (D/M ratio) and eNOS M/Total eNOS protein ratio (M/T ratio) in the frontal cortex (FC) (**A**–**C**) and hippocampus (**D**–**F**). Doses of the compounds are indicated in parenthesis in mg/kg. The data are presented as means ± SEM. Statistical analysis (SA) was performed using one-way ANOVA followed by Tukey’s multiple comparison. Statistical analysis for FC: (**A**) F_(2.25)_ = 23.26, *p* < 0.0001; (**B**) F_(2.25)_ = 13.65, *p* < 0.0001 (CDPPB) and F_(2.25)_ = 8.22, *p* < 0.001 (LY487379); (**C**) F_(2.25)_ = 15.65, *p* < 0.0001 (CDPPB) and F_(2.25)_ = 2.964, *p* < 0.06 (LY487379). Statistical analysis for hippocampus: (**D**) F_(2.25)_ = 7.8, *p* < 0.002 (CDPPB) and F_(2.25)_ = 10.07, *p* = 0.0006 (LY487379); (**E**) F_(2.25)_ = 6.007, *p* < 0.05 (CDPPB) and F_(2.25)_ = 13.82, *p* < 0.0001 (LY487379) and (**F**) F_(2.25)_ = 5.13, *p* < 0.01 (CDPPB ) and F_(2.25)_ = 7.44, *p* < 0.002 (LY487379. At least # *p* < 0.05, * *p* < 0.03, ** *p* < 0.01 and **** *p* < 0.0001 vs. control.

MK-801 administration decreased the eNOS D/M ratio and increased the eNOS M/T ratio in the FC. A decrease in the eNOS D/M ratio was observed in the hippocampus (Figure 2).

**Figure 3:**
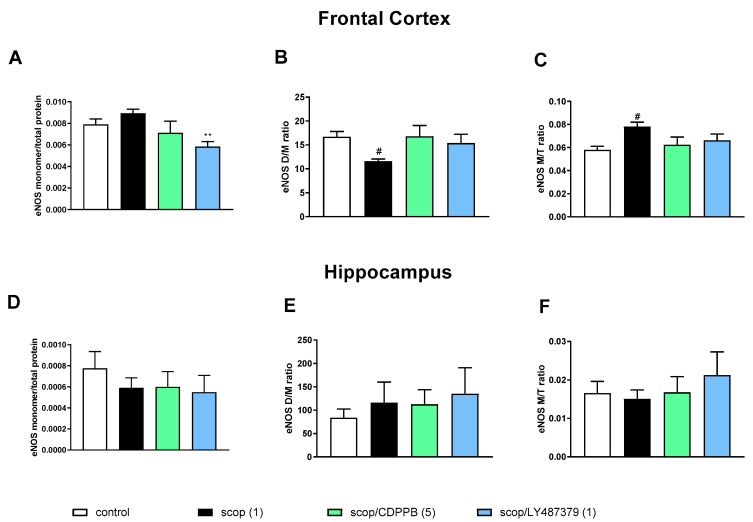
The impact of acute administration of scopolamine, CDPPB and LY487379 on eNOS monomer (M) content, eNOS dimer (D)/monomer (M) protein ratio (D/M ratio) and eNOS M/Total eNOS protein ratio (M/T ratio) in the frontal cortex (FC) (**A**–**C**) and hippocampus (**D**–**F**). Doses of the compounds are indicated in parenthesis in mg/kg. The data are presented as means ± SEM. Statistical analysis was performed using one-way ANOVA followed by Tukey’s multiple comparison. Statistical analysis for FC: (**A**) F_(2.25)_ = 12.46, *p* = 0.0002; (**B**) F_(2.25)_ = 11.16, *p* < 0.005 and (**C**) F_(2.25)_ = 4.22, *p* < 0.02. No statistically significant effects were observed in hippocampus. At least # *p* < 0.02, ** *p* < 0.008 vs. control. No statistical differences in subfigures (**D**–**F**).

Administration of scopolamine decreased the eNOS D/M ratio and increased the eNOS M/T ratio in FC. No changes were observed in the hippocampus (Figure 3).

The administration of CDPPB at the top dose further deepened the MK-801-induced effect significantly, increasing eNOS monomer content and the eNOS M/T ratio and decreasing the eNOS D/M ratio in the FC. LY487379 had no effect on MK-801-induced impairments (Figure 2).

In the hippocampus, both investigated compounds increased eNOS monomer content and the eNOS M/T ratio and decreased the eNOS D/M ratio when compared both to the control and the MK-801-treated group (Figure 2).

In the FCs of the scopolamine-treated groups, the administration of CDPPB and LY487379 decreased eNOS monomer content and increased the eNOS D/M ratio (Figure 3). No changes were observed after compound administration in the hippocampus (Figure 3).

Detailed statistics are indicated under each figure.

#### 2.2.2. Chronic Administration

**Figure 4:**
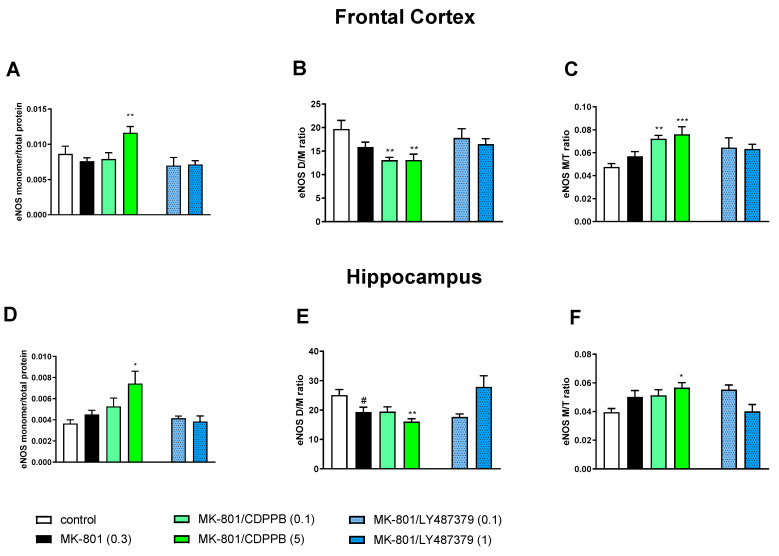
The impact of chronic (14 days) administration of MK-801, CDPPB and LY487379 on eNOS monomer (M) content, eNOS dimer (D)/monomer (M) protein ratio (D/M ratio) and eNOS M/Total protein ratio (M/T ratio) in the frontal cortex (FC) (**A**–**C**) and hippocampus (**D**–**F**). Doses of the compounds are indicated in parenthesis in mg/kg. The data are presented as means ± SEM. Statistical analysis was performed using one-way ANOVA followed by Tukey’s multiple comparison. Statistical analysis for FC: (**A**) F_(3.32)_ = 4.6, *p* < 0.01; (**B**) F_(3.32)_ = 6.4, *p* < 0.001 and (**C**) F_(3.32)_ = 8.2, *p* < 0.0003. Statistical analysis for hippocampus: (**D**) F_(3.29)_ = 3.7, *p* < 0.02; (**E**) F_(3.29)_ = 3.9, *p* < 0.01 (CDPPB) and F_(3.29)_ = 3.9, *p* < 0.01 (LY487379) and (**F**) F_(3.29)_ = 3.48, *p* < 0.02 (CDPPB) and F_(3.29)_ = 3.8, *p* < 0.02 (LY487379). A least # *p* < 0.05, * *p* < 0.03, ** *p* < 0.003 and *** *p* < 0.000 vs. controls.

MK-801 administration decreased the eNOS D/M ratio and increased the eNOS M/T ratio in the FC, but the effect did not reach statistical significance (Figure 4). A statistically significant decrease in the eNOS D/M ratio was observed in the hippocampus, as well as an increase in the eNOS M/T ratio that was not statistically significant (Figure 4).

**Figure 5:**
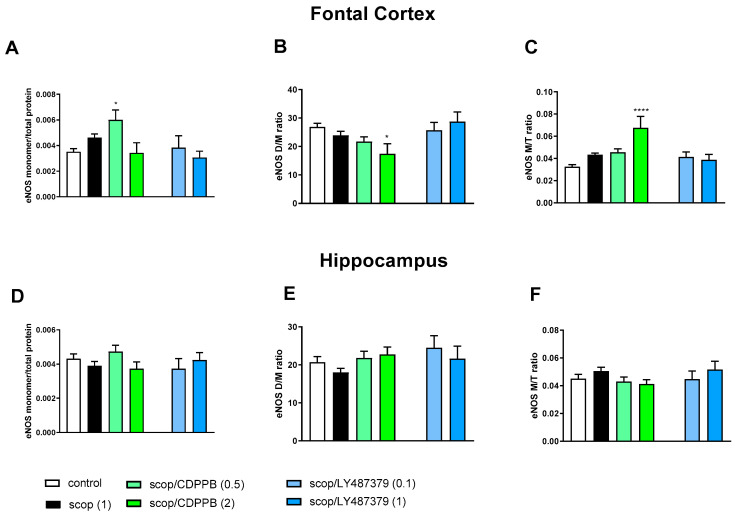
The impact of chronic (14 days) administration of scopolamine, CDPPB and LY487379 on eNOS monomer (M) content, eNOS dimer (D)/monomer (M) protein ratio (D/M ratio) and eNOS M/Total protein ratio (M/T ratio) in the frontal cortex (FC) (**A**–**C**) and hippocampus (**D**–**F**). Doses of the compounds are indicated in parenthesis in mg/kg. The data are presented as means ± SEM. Statistical analysis was performed using one-way ANOVA followed by Tukey’s multiple comparison. Statistical analysis in the FC: (**A**) F_(3.32)_ = 4.67, *p* = 0.008; (**B**) F_(3.32)_ = 3.78, *p* < 0.01 and (**C**) F_(3.32)_ = 8.4, *p* < 0.0003. No statistical changes in hippocampus were observed. At least * *p* < 0.01, **** *p* < 0.0001 vs. controls.

Administration of scopolamine increased eNOS monomer content, decreased the eNOS D/M ratio and increased the eNOS M/T protein ratio in the FC, but the effect did not reach statistical significance (Figure 4). No changes were observed in the hippocampus (Figure 5).

The administration of CDPPB at the top dose (5 mg/kg) further deepened the MK-801-induced effect, significantly increasing eNOS monomer content, and both doses of CDPPB decreased the eNOS D/M ratio and increased the eNOS M/T ratio in the FC. LY487379 had no effect on MK-801-induced impairments (Figure 4).

In the hippocampus, CDPPB at the higher dose of 5 mg/kg increased eNOS monomer content and the eNOS M/total protein ratio and decreased the eNOS D/M ratio when compared to the control group. LY487379 at a dose of 1 mg/kg reversed the MK-801-induced decrease in the eNOS D/M ratio (Figure 4).

In the FCs of scopolamine-treated groups, the administration of CDPPB at the dose 0.5 mg/kg increased eNOS monomer content and the eNOS M/T ratio, decreasing the eNOS D/M ratio. LY487379 had no effect in any of the investigated doses. No changes were observed in the hippocampus after both CDPPB and LY487379 administration (Figure 5).

Detailed statistics are indicated under each figure.

### 2.3. Novel Object Recognition

In all sets of experiments, in the acquisition trial, two-way ANOVA of exploration time of two identical objects (1 and 2) did not indicate any significant effects between groups.

In the retention trial, two-way ANOVA of the exploration time for familiar (F) and novel (N) objects in control vs. scopolamine- or MK-801-treated groups indicated that control animals explored the novel object significantly longer than the familiar object, and the ability to discriminate novel and familiar objects was abolished after scopolamine or MK-801 treatment. The data were analyzed using a two-way ANOVA followed by Tukey’s post-hoc comparison.

#### 2.3.1. Dose-Dependent Activity of CDPPB and LY487379 on Scopolamine-Induced Cognitive Deficits

**Figure 6:**
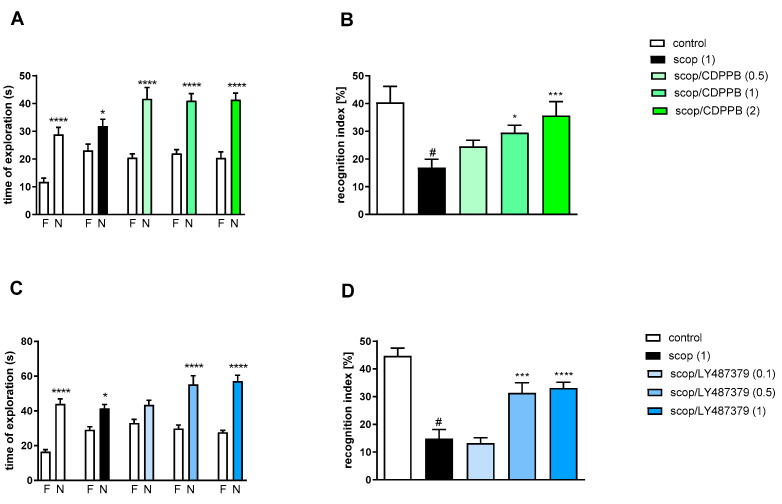
The effect of CDPPB (**A**,**B**) or LY487379 (**C**,**D**) on preventing scopolamine-induced cognitive impairment in novel object recognition test. Total time spent on exploring the familiar (F) or novel object (N) during the retention trial (**A**,**C**). Statistical analysis: (**A**) * *p* < 0.05 and **** *p* < 0.0001 vs. appropriate F times and (**C**) * *p* < 0.05 and **** *p* < 0.0001 vs. appropriate F times. Recognition index: (**B**) # *p* < 0.001 vs. control, * *p* < 0.02 and *** *p* < 0.0008 vs. scopolamine-treated group and (**D**) # *p* < 0.0001 vs. control, *** *p* < 0.0008 and **** *p* < 0.0001vs. scopolamine-treated group. Data are presented as the mean ± SEM. Doses in mg/kg are indicated in parentheses.

Two-way ANOVA of the exploration time for familiar (F) and novel (N) objects in scopolamine vs. scopolamine/CDPPB (0.5 mg/kg), scopolamine vs. scopolamine/CDPPB (1 mg/kg) or scopolamine vs. scopolamine/CDPPB (2 mg/kg) indicated that CDPPB administration prevented a scopolamine-induced decrease in N exploration time (F_(1.34)_ = 29.29; *p* < 0.04, F_(1.34)_ = 39.53; *p* < 0.0001 and F_(1.34)_ = 40.88; *p* < 0.0001, respectively) (Figure 6A).

Scopolamine administration significantly reduced the RI (t = 3.87, df = 19 and *p* < 0.001) (Figure 6B). A one-way ANOVA conducted across the treatment groups (with the scopolamine group as the reference group) on the RI showed statistically significant treatment differences (Figure 6B). The doses of 1 and 2 mg/kg reversed the scopolamine-induced disruption of novel object recognition (F_(3.39)_ = 5.78; *p* < 0.002). The dose of 0.5 mg/kg was ineffective.

Two-way ANOVA of the exploration time for familiar (F) and novel (N) objects in scopolamine vs. scopolamine/LY487379 (0.1 mg/kg), scopolamine vs. scopolamine/LY487379 (0.5 mg/kg) or scopolamine vs. scopolamine/LY487379 (1 mg/kg) indicated that LY487379 administration prevented a scopolamine-induced decrease in N exploration time (F_(1.34)_ = 25.95; *p* < 0.0001, F_(1.34)_ = 37.59; *p* < 0.0001 and F_(1.34)_ = 81.24; *p* < 0.0001, respectively) (Figure 6C).

Scopolamine administration significantly reduced the RI (t = 6.81, df = 17 and *p* < 0.001) (Figure 6D). One-way ANOVA conducted across the treatment groups (with scopolamine group as the reference group) on the RI showed statistically significant treatment differences (Figure 6D). The doses 0.5 and 1 mg/kg reversed the scopolamine-induced disruption of novel object recognition (F_(3.34)_ = 13.74; *p* < 0.0001). The dose of 0.1 mg/kg was ineffective.

#### 2.3.2. The Coadministration of CDPPB with spermineNONOate

**Figure 7:**
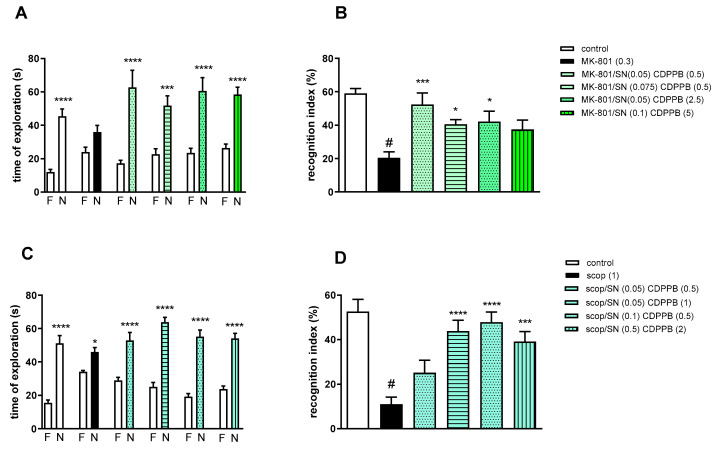
The effect of combined administration of spermineNONOate (SN) and CDPPB on preventing MK-801- (**A**,**B**) and scopolamine- (**C**,**D**) induced cognitive impairment in novel object recognition test. Total time spent on exploring the familiar (F) or novel object (N) during the retention trial (**A**,**C**) and recognition index (**B**,**D**). Statistical analysis (**A**) *** *p* < 0.0002 and **** *p* < 0.0001 vs. appropriate F times and (**C**) * *p* < 0.05 and **** *p* < 0.0001 vs. appropriate F times. Recognition index: (**B**) # *p* < 0.0001 vs. control, at least * *p* < 0.03 and *** *p* < 0.0006 vs. scopolamine-treated group and (**D**) # *p* < 0.0001 vs. control, *** *p* < 0.0005 and **** *p* < 0.0001 vs. scopolamine-treated group. Data are presented as the mean ± SEM. Doses in mg/kg are indicated in parentheses.

Two-way ANOVA of the exploration time for familiar (F) and novel (N) objects in MK-801 vs. MK-801/SN (0.05 mg/kg) and CDPPB (0.5 mg/kg), MK-801 vs. MK-801/SN (0.075 mg/kg) and CDPPB (0.5 mg/kg), MK-801 vs. MK-801/SN (0.05 mg/kg) and CDPPB (2.5 mg/kg) or MK-801 vs. MK-801/SN (0.1 mg/kg) and CDPPB (5 mg/kg) indicated that all combinations prevented MK-801-induced decrease in N exploration time (F_(1.28)_ = 24.46; *p* < 0.0001, F_(1.28)_ = 23.93; *p* < 0.001, F_(1.28)_ = 24.49; *p* < 0.0001 and F_(1.34)_ = 38.22; *p* < 0.0001, respectively) (Figure 7A).

MK-801 administration significantly reduced the RI (t = 8.29, df = 14 and *p* < 0.0001) (Figure 7B). One-way ANOVA conducted across the treatment groups (with the MK-801 group as the reference group) on the RI showed statistically significant treatment differences (Figure 7B). The combination of ineffective doses and moderate doses reversed the MK-801-induced disruption of novel object recognition (F_(4.35)_ = 4.74; *p* = 0.003). The combination of top doses was ineffective.

Two-way ANOVA of the exploration time for familiar (F) and novel (N) objects in scopolamine vs. scopolamine/SN (0.05 mg/kg) and CDPPB (0.5 mg/kg), scopolamine vs. scopolamine/SN (0.05 mg/kg) and CDPPB (1 mg/kg), scopolamine vs. scopolamine/SN (0.1 mg/kg) and CDPPB (0.5 mg/kg) or scopolamine vs. scopolamine/SN (0.5 mg/kg) and CDPPB (2 mg/kg) indicated that all combinations prevented scopolamine-induced decrease in N exploration time (F_(1.30)_ = 41.23; *p* < 0.0001, F_(1.30)_ = 112.5; *p* < 0.001, F_(1.30)_ = 89.72; *p* < 0.0001 and F_(1.30)_ = 86.27; *p* < 0.0001, respectively) (Figure 7C).

Scopolamine administration significantly reduced the RI (t = 7.32, df = 18 and *p* < 0.0001) (Figure 7D). One-way ANOVA conducted across the treatment groups (with the scopolamine group as the reference group) on the RI showed statistically significant treatment differences (Figure 7D). The combination of moderate and top doses reversed the scopolamine-induced disruption of novel object recognition (F_(4.35)_ = 10.76; *p* = 0.0001). The combination of subeffective doses was ineffective.

#### 2.3.3. The Coadministration of CDPPB with DETANONOate

**Figure 8:**
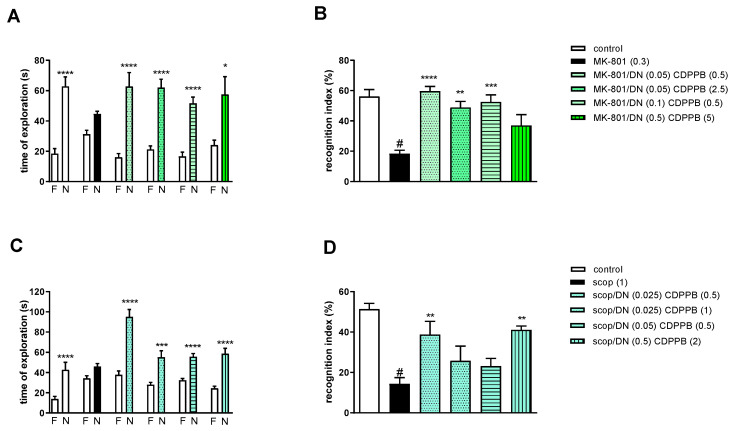
The effect of combined administration of DETANONOate (DN) and CDPPB on preventing MK-801- (**A**,**B**) and scopolamine- (**C**,**D**) induced cognitive impairment in novel object recognition test. Total time spent on exploring the familiar (F) or novel object (N) during the retention trial (**A**,**C**) and recognition index (**B**,**D**). Statistical analysis: (**A**) * *p* < 0.01 and **** *p* < 0.0001 vs. appropriate F times and (**C**) *** *p* < 0.0002 and **** *p* < 0.0001 vs. appropriate F times. Recognition index: (**B**) # *p* < 0.0001 vs. control, at least ** *p* < 0.001, *** *p* < 0.0003 and **** *p* < 0.0001 vs. scopolamine-treated group and (**D**) # *p* < 0.0001 vs. control, ** *p* < 0.005 vs. scopolamine-treated group. Data are presented as the mean ± SEM. Doses in mg/kg are indicated in parentheses.

Two-way ANOVA of the exploration time for familiar (F) and novel (N) objects in MK-801 vs. MK-801/DN (0.05 mg/kg) and CDPPB (0.5 mg/kg), MK-801 vs. MK-801/DN (0.05 mg/kg) and CDPPB (2.5 mg/kg), MK-801 vs. MK-801/DN (0.1 mg/kg) and CDPPB (0.5 mg/kg) or MK-801 vs. MK-801/DN (0.5 mg/kg) and CDPPB (5 mg/kg) indicated that all combinations prevented a MK-801-induced decrease in N exploration time (F_(1.22)_ = 23.39; *p* < 0.0001, F_(1.22)_ = 44.29; *p* < 0.001, F_(1.22)_ = 49.32; *p* < 0.0001 and F_(1.22)_ = 8.73; *p* < 0.01, respectively) (Figure 8A).

MK-801 administration significantly reduced the RI (t = 7.44, df = 8 and *p* < 0.0001) (Figure 8B). One-way ANOVA conducted across the treatment groups (with the MK-801 group as the reference group) on the RI showed statistically significant treatment differences (Figure 8B). The combination of ineffective doses and moderate doses reversed the MK-801-induced disruption of novel object recognition (F_(4.32)_ = 8.87; *p* = 0.0001). The combination of top doses was ineffective.

Two-way ANOVA of the exploration time for familiar (F) and novel (N) objects in scopolamine vs. scopolamine/DN (0.025 mg/kg) and CDPPB (0.5 mg/kg), scopolamine vs. scopolamine/DN (0.025 mg/kg) and CDPPB (1 mg/kg), scopolamine vs. scopolamine/DN (0.05 mg/kg) and CDPPB (0.5 mg/kg) or scopolamine vs. scopolamine/DN (0.5 mg/kg) and CDPPB (2 mg/kg) indicated that all combinations prevented a scopolamine-induced decrease in N exploration time (F_(1.30)_ = 41.23; *p* < 0.0001, F_(1.30)_ = 112.5; *p* < 0.001, F_(1.30)_ = 89.72; *p* < 0.0001 and F_(1.30)_ = 86.27; *p* < 0.0001, respectively) (Figure 8C).

Scopolamine administration significantly reduced the RI (t = 8.8, df = 14 and *p* < 0.0001) (Figure 8D). One-way ANOVA conducted across the treatment groups (with the scopolamine group as the reference group) on the RI showed statistically significant treatment differences (Figure 8D). The combination of subeffective and top doses reversed the scopolamine-induced disruption of novel object recognition (F_(4.35)_ = 5.07; *p* = 0.002). The combinations of moderate/low doses were ineffective.

#### 2.3.4. The Coadministration of LY487379 with spermineNONOate

**Figure 9:**
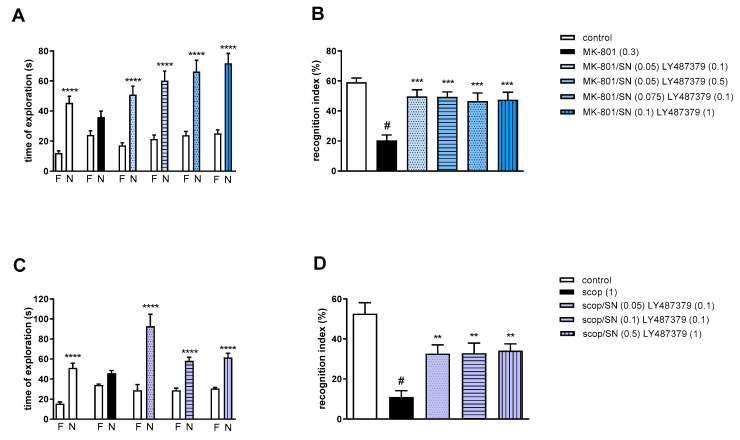
The effect of combined administration of spermineNONOate (SN) and LY487379 on preventing MK-801- (**A**,**B**) and scopolamine- (**C**,**D**) induced cognitive impairment in novel object recognition test. Total time spent on exploring the familiar (F) or novel object (N) during the retention trial (**A**,**C**) and recognition index (**B**,**D**). Statistical analysis: (**A**) **** *p* < 0.0001 vs. appropriate F times and (**C**) **** *p* < 0.0001 vs. appropriate F times. Recognition index: (**B**) # *p* < 0.0001 vs. control, at least *** *p* < 0.0008 vs. scopolamine-treated group and (**D**) # *p* < 0.0001 vs. control, at least ** *p* < 0.002 vs. scopolamine-treated group. Data are presented as the mean ± SEM. Doses in mg/kg are indicated in parentheses.

Two-way ANOVA of the exploration time for familiar (F) and novel (N) objects in MK-801 vs. MK-801/SN (0.05 mg/kg) and LY487379 (0.1 mg/kg), MK-801 vs. MK-801/SN (0.075 mg/kg) and LY487379 (0.1 mg/kg), MK-801 vs. MK-801/SN (0.05 mg/kg) and LY487379 (0.5 mg/kg) or MK-801 vs. MK-801/SN (0.1 mg/kg) and LY487379 (1 mg/kg) indicated that all combinations prevented a MK-801-induced decrease in N exploration time (F_(1.28)_ = 34.62; *p* < 0.0001, F_(1.28)_ = 35.35; *p* < 0.001, F_(1.28)_ = 33.18; *p* < 0.0001 and F_(1.34)_ = 45.71; *p* < 0.0001, respectively) (Figure 9A).

MK-801 administration significantly reduced the RI (t = 8.29, df = 14 and *p* < 0.0001) (Figure 9B). One-way ANOVA conducted across the treatment groups (with the MK-801 group as the reference group) on the RI showed statistically significant treatment differences (Figure 9B). All combinations reversed the MK-801-induced disruption of novel object recognition (F_(4.35)_ = 7.82; *p* = 0.0001).

Two-way ANOVA of the exploration time for familiar (F) and novel (N) objects in scopolamine vs. scopolamine/SN (0.05 mg/kg) and LY487379 (0.1 mg/kg), scopolamine vs. scopolamine/SN (0.1 mg/kg) and LY487379 (0.1 mg/kg) or scopolamine vs. scopolamine/SN (0.5 mg/kg) and LY487379 (1 mg/kg) indicated that all combinations prevented a scopolamine-induced decrease in N exploration time (F_(1.28_) = 30.56; *p* < 0.0001, F(1.28) = 63.19; *p* < 0.001 and F_(1.28)_ = 65.41; *p* < 0.0001, respectively) (Figure 9C).

Scopolamine administration significantly reduced the RI (t = 6.55, df = 14 and *p* < 0.0001) (Figure 9D). One-way ANOVA conducted across the treatment groups (with the scopolamine group as the reference group) on the RI showed statistically significant treatment differences (Figure 9D). All combinations reversed the scopolamine-induced disruption of novel object recognition (F_(3.28)_ = 7.4; *p* = 0.0008).

#### 2.3.5. The Coadministration of LY487379 with DETANONOate

**Figure 10:**
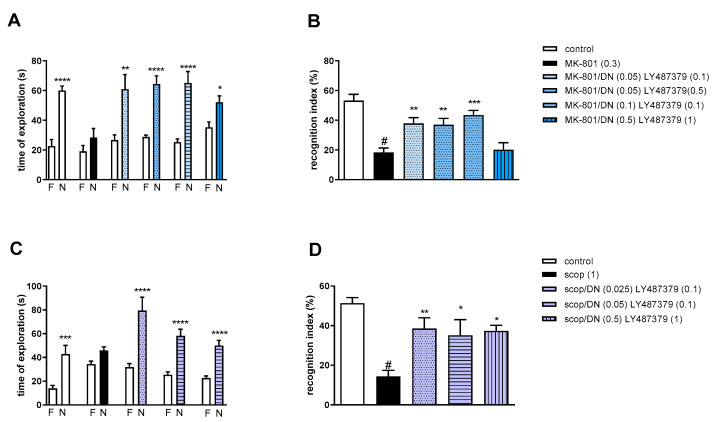
The effect of combined administration of DETANONOate (DN) and LY487379 on preventing MK-801- (**A**,**B**) and scopolamine- (**C**,**D**) induced cognitive impairment in novel object recognition test. Total time spent on exploring the familiar (F) or novel object (N) during the retention trial (**A**,**C**) and recognition index (**B**,**D**). Statistical analysis: (**A**) * *p* < 0.01, ** *p* < 0.001 and **** *p* < 0.0001 vs. appropriate F times and (**C**) *** *p* < 0.0004 and **** *p* < 0.0001 vs. appropriate F times. Recognition index: (**B**) # *p* < 0.0001 vs. control, at least ** *p* < 0.006 and *** *p* < 0.0002 vs. scopolamine-treated group and (**D**) # *p* < 0.0001 vs. control, at least * *p* < 0.02 and ** *p* < 0.008 vs. scopolamine-treated group. Data are presented as the mean ± SEM. Doses in mg/kg are indicated in parentheses.

Two-way ANOVA of the exploration time for familiar (F) and novel (N) objects in MK-801 vs. MK-801/DN (0.05 mg/kg) and LY487379 (0.1 mg/kg), MK-801 vs. MK-801/DN (0.05 mg/kg) and LY487379 (0.5 mg/kg), MK-801 vs. MK-801/DN (0.1 mg/kg) and LY487379 (0.1 mg/kg) or MK-801 vs. MK-801/DN (0.5 mg/kg) and LY487379 (1 mg/kg) indicated that all combinations prevented MK-801-induced decrease in N exploration time (F_(1.28)_ = 16.01; *p* < 0.001, F_(1.28)_ = 40.9; *p* < 0.001, F_(1.28)_ = 30.67; *p* < 0.0001 and F_(1.28)_ = 15.08; *p* < 0.01, respectively) (Figure 10A).

MK-801 administration significantly reduced the RI (t = 6.78, df = 13 and *p* < 0.0001) (Figure 10B). One-way ANOVA conducted across the treatment groups (with the MK-801 group as the reference group) on the RI showed statistically significant treatment differences (Figure 10B). The combinations of ineffective and moderate doses reversed the MK-801-induced disruption of novel object recognition (F_(4.35)_ = 8.47; *p* = 0.0001). The combination of top doses was not effective.

Two-way ANOVA of the exploration time for familiar (F) and novel (N) objects in scopolamine vs. scopolamine/DN (0.025 mg/kg) and LY487379 (0.1 mg/kg), scopolamine vs. scopolamine/DN (0.05 mg/kg) and LY487379 (0.1 mg/kg) or scopolamine vs. scopolamine/DN (0.5 mg/kg) and LY487379 (1 mg/kg) indicated that all combinations prevented a scopolamine-induced decrease in N exploration time (F_(1.28)_ = 22.37; *p* < 0.0001, F_(1.28)_ = 36.14; *p* < 0.0001 and F_(1.28)_ = 39.61; *p* < 0.0001, respectively) (Figure 10C).

Scopolamine administration significantly reduced the RI (t = 8.8, df = 14 and *p* < 0.0001) (Figure 10D). One-way ANOVA conducted across the treatment groups (with the scopolamine group as the reference group) on the RI showed statistically significant treatment differences (Figure 10D). All combinations reversed the scopolamine-induced disruption of novel object recognition (F_(3.28)_ = 4.6; *p* = 0.009).

## 3. Discussion

In these studies, the impact of metabotropic glutamate receptor ligands on eNOS expression in pharmacologically driven models of amnesia was investigated. The administration of mGlu_5_ PAM, CDPPB, and mGlu_2_ PAM, LY487379, enhanced MK-801- or scopolamine-induced endothelial dysfunction as manifested by increased eNOS monomer content and eNOS monomer/total protein ratio and a decreased eNOS dimer/monomer ratio in frontal cortices and hippocampi of mice brains. In subsequent investigations, it was proposed to counteract eNOS dysfunction via the simultaneous administration of NO releasers.

The potency of LY487379 and CDPPB to counteract MK-801-induced memory dysfunction was shown previously [41,42,43]. Here, the activity of the compounds on scopolamine-induced amnesia was demonstrated for the first time. The result confirms their procognitive power.

It is assumed that the activation of mGlu_2_ or mGlu_5_ receptors may prevent recognized causes of memory dysfunction, such as decreased NMDA-dependent currents on GABAergic neurons and subsequent overexpression of glutamate release from thalamocortical neurons [32,33,34]. Additionally, mGlu_5_ receptors regulate the glutamate–NO–cGMP pathway, which is crucial in long-term potentiation (LTP) and determines learning and memory processes [44,45,46,47]. The role of mGlu_2_ receptors in this process is less significant or less recognized but not excluded [47,48].

eNOS-related deficits including neurovascular endotheliopathy or BBB occur in schizophrenia or AD patients and have been observed in preclinical animal models [49,50]. Our previous and present results show that MK-801 or scopolamine administration promotes disruption of eNOS dimers to monomers [38] and the eNOS expression was impaired in an olfactory bulbectomy-induced model of AD [51]. To date, no other data related to these studies have been made available.

Our studies indicate that both mGlu activators can, to some extent, enhance eNOS monomer production or decrease the dimer/monomer ratio. CDPPB at a dose of 5 mg/kg exaggerates dimer disruption much above the control level. Although we previously showed that CDPPB counteracted eNOS dysfunction in the olfactory bulbectomy model [51], in the present schedule, at the higher dose of 5 mg/kg, this is questionable. The results are beyond expectations and indicate serious limitations related to the use of mGlu ligands.

Increased monomer content or a decreased dimer/monomer ratio promotes ROS or RNS production followed by oxidative or nitrosative stress, resulting in neuroinflammation. This was described in MK-801- or scopolamine-driven models and resembles the pathological changes linked with relevant brain disorders [52,53,54,55]. These further contribute to the cognitive and behavioral symptoms of schizophrenia or Alzheimer’s disease via mechanisms involving reduced cerebral perfusion, impaired homeostatic processes of cerebral microenvironment, harmful neuroimmune signals and toxic neuroinflammatory responses [2,10,14,15].

Therefore, the other approach to increasing the amount of bioavailable NO is to decrease the level of oxidative stress. Studies including newly developed acetylcholinesterase inhibitors with antioxidant properties revealed their antioxidant potential and reversal of cognitive deficits comparable to the standard donepezil drug used in treatment of AD [56,57].

To prevent endothelial dysfunction, which could develop along with the administration of mGlu PAMs and potentially aggravate the pathology or induce adverse effects, we proposed supplementing mGlu-based treatments with NO releasers.

A significant amount of research to date has indicated that NO donors induce an antiamnesic effect when administered alone [35,36,58,59,60,61]. The most commonly known NO donor, sodium nitroprusside, reversed MK-801- or ketamine-induced cognitive deficits in animal models [61,62] and effectively improved cognition in randomized double-blind placebo-controlled studies in schizophrenic patients [63]. The high risk of inducing adverse effects such as low blood pressure, cyanide toxicity and methemoglobinemia limits the use of the compound in humans [64].

NONOates (diazenium diolates) are a significant type of NO^•^ donor and are the result of exposing NO to a nucleophile, with the end product being flexible and predictable [65]. Studies concerning the activity of NONOates in animal models of brain disorders have indicated the potency of the compounds to decrease infarct size and prevent vasospasms caused by stroke in rodent models of ischemia [66,67]. Our recent studies on NONOates proved that the compounds are potent antiamnesic agents as well [35,36]. The potency of the compounds to prevent not only amnesia but also other symptoms accompanying brain disorders would constitute a great benefit. However, further investigation on this subject is needed.

Some reports have also suggested that spermine NONOate may have a unique pattern of NO release, which, for example, could modulate angiogenesis differentially [68,69]. Thus, regarding the use of a particular type of NO donor to improve cognition, its effectiveness and safety may depend on the pathology underlying the progression of dementia. It has to be remembered that the cognitive dysfunctions that accompany schizophrenia or depression can develop as a consequence of cardiovascular disorders and may result from impaired blood flow in the brain (vascular dementia) [70].

Summing up, the mutual supplementation of the antiamnesic activity of NONOates or mGlu activators potentiates the activity of each factor individually and may be less burdened with the induction of adverse effects, which could develop as a consequence of the compounds administered alone at top doses. The administration of top doses does not bring any additive results.

## 4. Materials and Methods

### 4.1. Animals

In all experiments, male albino Swiss mice (Charles River Laboratory, Sulzfeld, Germany) weighing between 20 and 25 g were used. All animals were kept at room temperature (22 ± 1 °C) with free access to standard chow diet and water, under a 12/12 light–dark cycle. Each experimental group consisted of at least 8 animals. The compounds were administered intraperitoneally, at a volume of 10 mL/kg. Animals were kept in conditions in accordance with EU Directive 2010/63/EU and subsequent regulations of the Polish Ministry of Agriculture and Rural Development.

### 4.2. Western Blotting

Fragments of brain, both FC and hippocampus, were ground in liquid nitrogen to a powder and transferred to an ice-cold RIPA lysis buffer (Cell Signaling Technology, Leiden, The Netherlands) supplemented with PMSF (1 mM) and a Protease Inhibitor Cocktail (Sigma-Aldrich, Darmstadt, Germany). Samples were vortexed and incubated on ice for 15 min. After the incubation period, the samples were centrifuged at 4 °C (12,000× *g* for 15 min) and the supernatants were collected. The protein concentration in the obtained supernatants was measured with the DC™ Protein Assay Kit II (Bio-Rad, Basel, Switzerland).

Samples were then diluted with a Laemmli buffer (Bio-Rad, Basel, Switzerland, Cat# 1610747) containing 2.5% β-mercaptoethanol. A 10 µL quantity of each sample containing 40 µg of total protein was loaded on 4–15% polyacrylamide gels (Bio-Rad, Basel, Switzerland) submerged in an ice-cold running buffer (Bio-Rad, Basel, Switzerland). Subsequently, low-temperature SDS-PAGE in an ice bath was performed.

In the next step, gels were imaged under UV light to enable the measurement of total protein levels. Proteins were transferred to PVDF membranes (Bio-Rad, Basel, Switzerland) using the Trans-Blot^®^ Turbo™ Transfer System (Bio-Rad, Basel, Switzerland). The membranes were blocked with 5% BSA (Sigma-Aldrich, Darmstadt, Germany) for 30 min and incubated with primary rabbit monoclonal anti-eNOS antibody (1:1000 dilution, Cell Signaling Technology, Leiden, The Netherlands) at 4 °C overnight. The next day, after extensive washing, membranes were incubated with a goat anti-rabbit IgG HRP-conjugated secondary antibody (1:2000 dilution, Cell Signaling Technology, Leiden, The Netherlands) at room temperature for 1 h. After incubation with VisiGlo™ HRP chemiluminescent substrate (VWR, Radnor, PA, USA), membranes were visualized using the ChemiDoc Imaging System (Bio-Rad, Basel, Switzerland).

Densitometric measurements of eNOS expression and total protein level were performed using Image Lab software v. 4.1 (BioRad, Basel, Switzerland). The density of the obtained bands of eNOS was first normalized to total protein levels established from gels and then the ratio was calculated. eNOS levels were analyzed as monomer/total protein ratio, dimer/monomer (D/M) ratio and monomer/total eNOS (M/T) ratios [71].

### 4.3. Novel Object Recognition

The NOR test was performed according to the previously described method [35,72]. Briefly, a black, plastic rectangular arena illuminated with a 355-lux bulb situated in a dark room was used for performing the habituation, training and test trials. During the two-day long habituation trial, mice were allowed to explore the arena in the absence of objects for 10 min per day. The next day, the training (T1) and test (T2) trials were performed. In both T1 and T2, animals were allowed to explore objects freely for 5 min. Throughout T1, two identical objects were used. In T2 (1 h later), one of those objects was replaced by a new one. Time spent on exploring (i.e., sniffing or touching) the familiar (TF) or novel object (TN) was measured by a trained observer, and then the recognition index was calculated for each mouse: [(TN − TF)/(TF + TN)] × 100.

### 4.4. Statistics

The data are presented as means ± S.E.M. Statistical analysis of the data was performed with GraphPad Prism 8.1.1. eNOS monomer, dimer/monomer (D/M) and monomer/total (M/total) ratio results were analyzed with the use of one-way ANOVA (or nonparametric analysis when the normal distribution was not met) followed by Dunnett’s post-hoc comparison. Statistical analysis of NOR results was performed according to previous studies [73]. For the acquisition and retention trial in NOR, the exploration data of familiar (1) vs. familiar (2) object or familiar (F) vs. novel (N) object within the treatment were analyzed using a two-way analysis of variance (ANOVA) followed by Tukey’s post hoc multiple comparison test as established. The analysis was performed for the following groups: control vs. MK-801, MK-801 vs. each MK-801/treatment, control vs. scopolamine and scopolamine vs. each scopolamine/treatment. The RI data were analyzed as follows: control vs. MK-801 (or scopolamine) groups were analyzed using Student’s *t*-test in order to validate the execution of the experiment, then the data were analyzed using one-way ANOVA across drug treatments (with MK-801 or scopolamine as reference groups) followed by Dunnett’s post hoc multiple comparison test. *p* values were considered as significant when *p* was at least * <0.05, ** <0.01, *** <0.001 and **** <0.0001.

## 5. Conclusions

The prevention or treatment of dementia constitutes the challenge of our times. Stress, sleep disturbances and an unbalanced lifestyle result in an increasing number of psychiatric disorders and cognitive dysfunctions. The number of patients diagnosed with neurodegenerative disorders, with AD at the forefront, is also increasing.

Schizophrenia is diagnosed in early adulthood, excluding the individual from normal functioning in the majority of cases. Developing cognitive decline hampers professional work.

Presently, no effective drugs to treat cognitive decline are available. mGlu_2_ or mGlu_5_ receptor ligands could be proposed as a solution; however, these studies indicate that the administration of the compounds alone could trigger eNOS dysfunction and enhance neuroinflammation. Therefore, to avoid this risk, the administration of the compounds at minimal possible doses is recommended. Supplementation with NO releasers could be proposed. To date, no data on the activity of the investigated ligands in terms of eNOS expression have been shown. Our results are pioneering in the field, and our prospective studies will endeavor to further explore this area, with particular focus on neuroinflammation and ROS/RNS production.

The use of pharmacologically driven models constitutes a limitation of this study. To confirm the results, the use of the other animal models such as a developmental model of schizophrenia or transgenic mouse models of AD, based on APP gene mutations, could be used. Also, the use of other compounds would be of interest, especially biased agonist or allosteric modulators of metabotropic glutamate receptor 5, which could differentially influence signaling to distinct transducers and pathways [74,75].

## Figures and Tables

**Table 1:** Pharmacological properties, full names, sources and solvents of the compounds used in the studies.

Compound	Properties		
spermineNONOate (Tocris, Bristol, UK) *(Z)-1-[N-[3-aminopropyl]-N-[4-(3-aminopropylammonio)butyl]-amino]diazen-1-ium-1,2-diolate*	Fast NO releaser t_1/2_ ≈ 39 min (37 °C, pH = 7.4) or 230 min (22–25 °C, pH = 7.4) *1 mole of spermine NONOate generates 2 moles of NO*	0.9% NaCl	[37]
DETANONOate (Tocris, Bristol, UK) *(Z)-1-[N-(2-aminoethyl)-N-(2-ammonioethyl)amino]diazen-1-ium-1,2-diolate*	Slow NO releaser t_1/2_ ≈ 20 h (37 °C, pH = 7.4) or 56 h (22–25 °C, pH = 7.4) 1 mole of DETA NONOate generates 2 moles of NO	0.9% NaCl	[37]
MK-801 (Tocris, Bristol, UK) *(5*S*,10*R*)-(+)-5-Methyl-10,11-dihydro-5*H*-dibenzo[*a*,*d*] cyclohepten-5,10-imine maleate*	Selective and noncompetitive NMDA antagonist; K_i_ = 37.2 nM	0.9% NaCl	[38]
Scopolamine hydrobromide (Abcam, Cambridge, UK) *(α,*S*)-α-(Hydroxymethyl) benzeneacetic acid (1α,2β,4β,5α,7β)-9-methyl-3-oxa-9-azatricyclo [3.3.1.02,4]non-7-yl ester*	Nonselective muscarinic antagonist	0.9% NaCl	[38]
LY487379 (Tocris, Bristol, UK) *N-(4-(2-Methoxyphenoxy)phenyl)-N-(2,2,2-trifluoroethylsulfonyl) pyrid-3-ylmethylamine hydrochloride*	mGlu_2_ positive allosteric modulator; EC_50_ = 1.7 μM	0.9% NaCl	[39]
CDPPB (Abcam, Cambridge, UK) *3-Cyano-*N*-(1,3-diphenyl-1*H*-pyrazol-5-ylbenzamide*	mGlu_5_ positive allosteric modulator; EC_50_ values are 10 and 20 nM for human and rat, respectively	10% Tween 80	[40]

**Table 2:** The administration schedule of the combined administration of mGlu receptor ligands—mGlu_2_ PAM LY487379 and mGlu_5_ PAM-CDPPB—with fast (DETANONOate) and slow (spermineNONOate) releaser. Doses are indicated in parenthesis as mg/kg.

	MK-801 (0.3)	Scopolamine (1)
Ineffective doses	SpermineNONOate (0.05) + CDPPB (0.5) SpermineNONOate (0.05) + LY487379 (0.1) DETANONOate (0.05) + CDPPB (0.5) DETANONOate (0.05) + LY487379 (0.1)	SpermineNONOate (0.05) + CDPPB (0.05) SpermineNONOate (0.05) + LY487379 (0.1) DETANONOate (0.025) + CDPPB (0.5) DETANONOate (0.025) + LY487379 (0.1)
Low/moderate doses	SpermineNONOate (0.075) + CDPPB (0.5) SpermineNONOate (0.05) + CDPPB (2.5) SpermineNONOate (0.05) + LY487379 (0.5) SpermineNONOate (0.75) + LY487379 (0.1) DETANONOate (0.05) + CDPPB (2.5) DETANONOate (0.1) + CDPPB (0.5) DETANONOate (0.05) + LY487379 (0.5) DETANONOate (0.1) + LY487379 (0.1)	SpermineNONOate (0.1) + CDPPB (0.5) SpermineNONOate (0.05) + CDPPB (1) SpermineNONOate (0.1) + LY487379 (0.1) DETANONOate (0.05) + CDPPB (0.5) DETANONOate (0.025) + CDPPB (1) DETANONOate (0.05) + LY487379 (0.1)
Top doses	SpermineNONOate (0.1) + CDPPB (5) SpermineNONOate (0.1) + LY487379 (1) DETANONOate (0.5) + CDPPB (5) DETANONOate (0.5) + LY487379 (1)	SpermineNONOate (0.5) + CDPPB (2) SpermineNONOate (0.5) + LY487379 (1) DETANONOate (0.5) + CDPPB (2) DETANONOate (0.5) + LY487379 (1)

## Data Availability

Data can be made available per request.
